# The XIX century smallpox prevention in Naples and the risk of transmission of human blood-related pathogens

**DOI:** 10.1186/s12967-015-0400-9

**Published:** 2015-01-27

**Authors:** Franco Maria Buonaguro, Maria Lina Tornesello, Luigi Buonaguro

**Affiliations:** Department of Experimental Oncology, Division of Molecular Biology and Viral Oncology, Istituto Nazionale per lo Studio e la Cura dei Tumori – IRCCS “Fondazione Pascale”, Via Mariano Semmola 142, 80131 Napoli, Italy

## Abstract

Vaccines are the most successful strategy developed in Medicine to prevent and even eradicate the most dreadful epidemic infectious diseases. The history of smallpox vaccination in Naples is quite unique. Although Galbiati established the retro-vaccination (1803) and developed the “calf” lymph vaccine, recognized and implemented since 1864 as the optimal smallpox vaccine in the following hundred years, Naples general population was mainly vaccinated with “human” lymph from abandoned children until 1893. Mini-epidemics of syphilis and serum hepatitis were periodically reported as results of arm-to-arm procedure. The risk of transmission of blood-related pathogens was higher in Naples where >80% of abandoned children, used as repository of cowpox virus, were dying in their first year of life. Recent vaccinology standards finally eliminated the risk of adventitious contaminating pathogens. Implementation of hepatitis B vaccination since 1991 eventually contributed to current HBV prevalence in Campania region <1%, within the range of the European Countries.

The history of smallpox prevention in Southern Italy is very peculiar, being characterized by the development and implementation of the first calf lymph vaccine and by a late banning of the arm to arm vaccination.

Prevention of smallpox (Variola major) represents the first preventive strategy of an infectious disease in Medicine. The variolation procedure, developed in China since the XVI century with transfer of crusts from minor lesions of infected patients to healthy subjects [1], was introduced in England in1717 by Lady Mary Wortley Montagu [2]. The further critical scientific achievements were the observation by John Fewster (1765) that bovine cowpox was able to prevent human smallpox [3] and the standardization (1798) of the cowpox vaccination by Edward Jenner [4]. Since Jenner’s report several strategies were introduced in Europe to contain the terrible disease, whose epidemics decimated entire communities. In particular, given the sporadic disease in cows [5], cowpox crusts were distributed to different countries to perform vaccinations. Within this approach Joseph Marshall and John Walker were sent to Gibraltar and Malta, and subsequently to Egypt and Sicily. In Palermo the first vaccination was performed on March 14^th^ 1801[6]. Calcagni meticulously describes vaccination procedures, number of vaccinees, local reaction and pustules, as well as number of vaccinated children used to propagate the disease, which was locally transferred arm-to-arm from a vaccinated child to the others [7]. Concomitantly other approaches were taken to have a local supply of cowpox material. Luigi Sacco, in Milan, identified cows with lesions similar to those described by Jenner and vaccinated human subjects with cowpox crusts taken directly from cows raised near Varese in Lombardy [7]. Gennaro Galbiati and Michele Troja, in Naples, introduced the retro-vaccination. In 1803 they were able to prove that cowpox could be transferred from cowpox-vaccinated people to cows (retro-vaccination) and the recovered calf lymphatic material was very effective for further vaccination of humans [7]. Galbiati, head of the Gynecology unit at the Hospital for the Incurables and member of the Accademia Medico-Cerusica (Academy of Medicine and Surgery) of Naples, founded by Murat during the Napoleonic Kingdom of Naples, fully developed the system and in 1810 accurately describes the three major reasons to prefer the animal vaccination: the possibility to expand the production of vaccine, the recovery of pathogenicity lost in the human transfer, and mainly the removal of human contaminating pathogens not transferred to cows [8,9]. In the same year, persuaded of the advantages of the lymph calf vaccine, he established his own vaccine production facility [10]. The animal vaccination was mainly used by the aristocrats and the King family, along with some of the most prestigious medical colleagues, including Cotugno, Villari and Sementini. The cowpox production was continued by Galbiati’s students, Ferdinando Palasciano and Giuseppe Negri, who substituted Galbiati at his death in 1844. In 1849 Negri, was finally able to have some cows from the royal park for the monthly inoculation of the cowpox and the continuous production of vaccine, used for the court needs and the remaining for the general population. Following the establishment of the unified Italian Kingdom (1861), Negri continued the animal vaccine production at his own expenses [10] and after the Congrès Medical de Lyon (1864), established further production facilities in Paris and Lyon [11].

However, regardless the scientific advancements achieved by Galbiati, the animal vaccine was never fully accepted in Naples, actually a very strong controversy began with those physicians appointed at Vaccine Committee [12], who tried even to have a law for the prohibition of the animal calf lymph vaccination and finally established a journal *La Biblioteca Vaccinica* (The vaccine library) to constantly discredit Galbiati’s institution [10].

The Vaccine Committee always preferred the most popular arm-to-arm vaccination procedure, very fashionable since the 1803–06 expedition de la vacuna financed by King Carlos IV of Spain for the vaccination of thousands of people living in the New World and Asia [13]. For the vaccination program Francisco Xavier De Balmis shipped out of Spain with 22 orphan children sequentially arm-to-arm immunized throughout the campaign, creating an effective living chain of readily available live vaccine. In Naples vaccine donors for the arm-to-arm cowpox vaccination prevalently were the vaccinated children from the local orphanage “La Real Casa dell’Annunziata di Napoli” (the Royal House of our Lady of Annunciation) of Naples. The Royal House, built in 1330, became in 1650 an official Orphanage where the abandoned “exposed” children, offered to our Lady, were considered the children “protected by” the Virgin Mary. The children were vaccinated few hours after the arrival in the orphanage and the “most healthy” were used as vaccine producers for the extraction of the lymphatic vaccine as well as for the arm-to-arm inoculation. The children of the Orphanage were the actual repository of the “humanized” cowpox for the entire province, for the public institutions and for the militaries [14]. Unfortunately it was not fully recognized the major risk of diffusion of human diseases in particular syphilis, hepatitis, tuberculosis, reported in several occasions. This risk was much greater with those “vaccinifer” children, given that infectious diseases were more frequent in the illegitimate “exposed” children, whose syphilis prevalence was >10-fold higher in comparison to legitimate children of the Italian general population. The frequency of such event was sufficiently high to justify the identification of a specific nosographic disease: the vaccine’s syphilis [14]. Local syphilis mini-epidemics were also reported, as the first in Rivalta (1861) with the resulting infection of 44 out of 63 vaccinated children and some of their nurses [15,16]. The arm-to-arm vaccination accidentally transferred also the less recognized hepatitis B, whose first epidemic occurred in Bremen in 1883 following the cowpox vaccination of 1,289 shipyard employees. 191 of the vaccinated workers became ill with jaundice and diagnosed with serum hepatitis [17]. All such reports pressed the scientific community to gradually replace the human vaccination with the calf lymph vaccination, which following the Lyon Meeting of 1864 [11] was immediately arranged by Chambon and Lanoix in Paris and gradually extended through France (Depaul, 1867). Belgium adopted it in 1865, Germany by 1884, and The Netherlands followed suit. The arm-to-arm vaccination was finally banned in Great Britain in 1898. In the same year in USA the Vaccination Act banned arm-to-arm vaccination, with the full introduction of calf vaccine, produced since 1876 by the New York Health Department, and later by Wyeth as the Dryvax vaccine up to 1980.

In Naples the human lymph was substituted by the calf lymph vaccine only in1893, following a major debate and concern on the Annunziata Orphanage. The syphilis, known as the Naples disease since the first epidemic of 1494, was decimating >80% of the illegitimate orphans in their first year of life and the risk of transmission with the humanized vaccine was extremely high [18]. No documented reports are available on the transmission frequency of other infectious pathogens, which must have been high as well, considering the sexual promiscuity, the congenital transmission and the systemic diffusion of the pathogens [Table 1 & Figure 1]. The difficulty to identify and characterize other transmissible diseases was also due to pleomorphism of syphilis, described as “the great imitator” by Sir William Osler. The involvement (in the secondary stage) of several organs, including liver, would have masked the transmission of other pathogens in absence of appropriated diagnostic tools.

**Table 1:** **Prevalent causes of death of legitimate and illegitimate children in the analyzed calendar years**

**Causes of death**	Legitimate children*					Illegitimate/exposed children*				
**Calendar Year**	**1887**		**1888**		**1889**	**1887**		**1888**		**1889**
**Preterm birth and congenital malformations**	**46.0**		**49.4**		**44.9**	**74.7**		**78.0**		**73.1**
**Enteritis and diarrhea**	**33.1**		**35.4**		**34.3**	**47.4**		**45.6**		**44.9**
**Gastritis**	**---**		**---**		**---**	**---**		**---**		**---**
**Syphilis**	**0.5**		**0.5**		**0.5**	**8.0**		**9.1**		**9.8**
**Calendar Year**	**1896**	**1897**	**1898**	**1899**	**1900**	**1896**	**1897**	**1898**	**1899**	**1900**
**Preterm birth and congenital malformations**	**43.6**	**40.5**	**41.8**	**40.3**	**44.9**	**81.0**	**69.2**	**73.6**	**71.9**	**67.7**
**Enteritis and diarrhea**	**39.5**	**38.5**	**43.0**	**36.5**	**44.7**	**60.5**	**54.3**	**58.7**	**52.1**	**37.5**
**Gastritis**	**2.7**	**3.1**	**3.3**	**3.2**	**3.6**	**4.9**	**6.1**	**6.7**	**7.6**	**8.0**
**Syphilis**	**0.6**	**0.6**	**0.5**	**0.6**	**0.6**	**12.0**	**10.6**	**12.2**	**13.4**	**10.4**

*Number of children death in the first year of life per 1000 children born alive.

(English version of Figure 1, from page 57 of Ref [14]).

**Figure 1:**
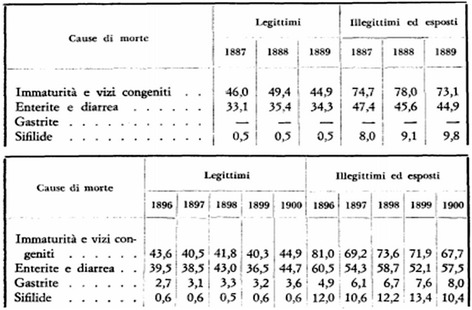
**Number of children death in the first year of life/1000 children born alive.** (Original table in Italian at the page 57 of Ref [14]).

For such reason, as frequently it happens in history, a peculiar combination of events made the first town to develop a calf lymph vaccine, one of the last town to definitely ban the “humanized” cowpox vaccination, only when the risk of transmission of human blood-related infections became enormously high. To evaluate the role of the humanized cowpox and the arm-to-arm procedure on the current high prevalence of blood-related infections present in the Southern part of Italy, including those (i.e. HBV and HCV) at high risk of cancer progression, would be fully speculative in absence of any documented data. It is however peculiar the very high prevalence of HBV (>10%) and HCV (>30%) in subjects over 65 years of age, with an annual progression rate of HCV infection to HCC >7.0% [19]. Moreover, the HCV prevalence <10% in the 30–35 years age group, strongly supports a cohort effect in which the risk for HCV infection was higher in the distant past [19].

## Conclusions

The history of small pox vaccination in Naples is extremely peculiar with two major aspects (a) the development of the retro-vaccination, which became popular in the rest of the world more than 65 years later; (b) the use of the “humanized” cowpox vaccination from subjects at high risk of infectious diseases. The role on such events of the political issues and the contrasts between the French-appointed Galbiati and Troja’s family and the Spanish-supported physicians will be unlikely clarified. It is however extremely strange that neither streets nor Hospital have been dedicated to Galbiati’s memory and his name has been completely forgotten. Luckily, the infectious risks of vaccination, including those reported in this article, are past history. The different manufacture and properties of vaccines, currently produced in pathogen-free environment and based on pathogen sub-units, not able to induce any disease, along with blood screening and use of disposable tools, on the contrary, have strongly contributed to the drastic decrease of iatrogenic infections also in Southern Italy. In particular the hepatitis B vaccination, mandatory since 1991, has drastically reduced the HBV prevalence in the Campania region to <1% within the range of the rest of Italy and the majority of the Western Countries [20].

The high safety of the current immune therapeutic compounds along with their unquestionable efficacy are properly crediting vaccines as one of the most effective treatment of the medical history.
